# Multi-stage progressive detection method for water deficit detection in vertical greenery plants

**DOI:** 10.1038/s41598-024-60179-3

**Published:** 2024-04-26

**Authors:** Fei Deng, Xuan Liu, Peng Zhou, Jianglin Shen, Yuanxiang Huang

**Affiliations:** 1 College of Computer Science and Cyber Security, Chengdu University of Technology, Chengdu, 610059 China; 2 Sichuan Tianyi Ecological Garden Group Co., Ltd., No. 1 Keyuan South Road, High-tech Zone, Chengdu, 610093 Sichuan China

**Keywords:** Vertical greening plant, Deep learning, Object detection, Multi-stage, Swin transformer, Self-attention, Computer science, Plant sciences

## Abstract

Detecting the water deficit status of vertical greenery plants rapidly and accurately is a significant challenge in the process of cultivating and planting greenery plants. Currently, the mainstream method involves utilizing a single target detection algorithm for this task. However, in complex real-world scenarios, the accuracy of detection is influenced by factors such as image quality and background environment. Therefore, we propose a multi-stage progressive detection method aimed at enhancing detection accuracy by gradually filtering, processing, and detecting images through a multi-stage architecture. Additionally, to reduce the additional computational load brought by multiple stages and improve overall detection efficiency, we introduce a Swin Transformer based on mobile windows and hierarchical representations for feature extraction, along with global feature modeling through a self-attention mechanism. The experimental results demonstrate that our multi-stage detection approach achieves high accuracy in vertical greenery plants detection tasks, with an average precision of 93.5%. This represents an improvement of 19.2%, 17.3%, 13.8%, and 9.2% compared to Mask R-CNN (74.3%), YOLOv7 (76.2%), DETR (79.7%), and Deformable DETR (84.3%), respectively.

## Introduction

Vertical greenery is one of the important forms of urban greening and an effective way to improve the urban ecological environment and enrich urban green landscape^1,2^. While there are many factors that influence the growth of greenery plants, in practical scenarios, water deficit is the primary reason leading to the wilting and death of greenery plants^3^, highlighting the need for timely and accurate detection of water deficiency in vertical greenery plants. Water deficit detection involves examining aspects such as the color, size, and surface features of plant leaves^4,5^. Based on the detection results, maintenance personnel can assess the overall growth status of plants, pinpoint the specific locations of water-deficient plants, and promptly implement corresponding preventive measures^6^. Simultaneously, understanding the general patterns of water requirements of greenery plants at different growth stages can provide a basis for decision-making in scientific planting and management.

In recent years, with the rapid development of deep learning technology in the fields of computer vision and image processing^7^, intelligent object recognition and detection have gained significant attention. In 2014, Girshick et al.^8^ first proposed the R-CNN object detection algorithm based on deep learning, which initially generates region proposals through selective search^9^, and then classifies and regresses the targets in these regions. Algorithms like Fast R-CNN^10^, Faster R-CNN^11^, and Mask R-CNN^12^ have evolved from this approach, belonging to the category of two-stage object detection algorithms based on region proposals^13^. Another type of algorithm is the single-stage object detection algorithms primarily represented by YOLO^14^ and SSD^15^ , which involve regression-based methods, eliminating the region proposal generation process^16^. These algorithms treat the entire object detection task as a regression task with a single set of parameters, directly regressing the labels and positions of objects, significantly enhancing detection efficiency.

Subsequently, the Transformer based on self-attention mechanism achieved outstanding performance in the field of NLP^17^. Due to its powerful representational capacity, scholars gradually began to apply it to the field of image processing. In 2020, Dos et al.^18^ first applied Transformer to the field of image classification, proposing the Vision Transformer (ViT). In 2021, Liu et al.^19^ introduced a Swin Transformer based on shifted window and hierarchical representation. This model incorporates features from different regions into self-attention computation through shifted windows, thereby expanding the receptive field of the window. Furthermore, it improves the computational efficiency of cross-window connections through a unique masking mechanism, resulting in a linear relationship between computational complexity and image size. Due to the better balance between accuracy and efficiency achieved by the Swin Transformer, it can serve as a universal backbone for various downstream tasks in computer vision such as image classification, segmentation, and object detection. Subsequently, in 2022, Wu et al.^20^ proposed an end-to-end multi-stage edge computing driven detection framework for enhancing and detecting objects in low-light images, greatly improving detection performance under strong and weak lighting conditions. These advancements provide theoretical support for our multi-stage detection method.

Although mainstream object detection algorithms have achieved satisfactory results on various public datasets, there are limitations in the practical application of detecting water deficit in vertical greenery plants^21,22^. On the one hand, the accuracy of these algorithms heavily relies on the overall quality of the images being detected. When encountering low-quality images such as those with low light at night or motion blur, or when there are complex background occlusions, the detection accuracy significantly decreases, leading to problems such as missed detections and false alarms. On the other hand, the purpose of detecting water-deficient plants is to comprehensively assess and analyze the growth status of plants in the area, requiring the calculation of the proportion of all water-deficient plants to the actual detection area. Traditional single-object detection algorithms cannot exclude background regions, making it impossible to perform effective data analysis. Therefore, overcoming these algorithm limitations remains a major challenge to be addressed.

To achieve this, we have developed a multi-stage progressive detection method based on Swin Transformer^19^. By filtering low-quality images and applying black masks to non-vertical greenery areas, we conduct water deficit plant detection and result analysis. This overcomes the drawbacks of existing object detection algorithms, which have low detection accuracy and cannot meet the demands of practical detection scenarios. Additionally, to enhance detection efficiency, a universal visual backbone, Swin Transformer, is employed in each stage to reduce network computation and accelerate image inference speed. Furthermore, multi-scale global features are extracted through a self-attention mechanism with a moving window to further enhance detection accuracy. In the comparative experiments, the multi-stage progressive detection method achieved an mAP of 92.4% on the vertical greenery dataset, significantly outperforming conventional object detection algorithms, further demonstrating the superiority of this approach.

The main contributions of this work are:

(1) We propose a multi-stage progressive detection method for the detection and analysis of water deficit in vertical greenery plants. This method combines three different types of networks: image classification, semantic segmentation, and object detection, to overcome the limitations of traditional single-object detection algorithms that cannot be applied to practical engineering problems.

(2) Swin transformer is adopted as the backbone network in each stage, with self-attention computations confined to non-overlapping local windows, thereby significantly reducing the additional computational burden brought by multiple stages. Meanwhile, the residual concept is introduced into some Swin Transformer Blocks to prevent the model from overfitting.

## Materials and methods

### Multi-stage progressive detection architecture

In practical detection scenarios, vertical greenery images are obtained by fixed cameras at regular intervals. During the shooting process, they inevitably suffer from external weather disturbances such as low light at night, rain, snow, and wind. At the same time, to capture greenery plants at different positions, the camera needs to continuously adjust its shooting direction and focus. These factors can lead to various noise and blur issues; for instance, improper focusing of the camera can result in defocus blur^23^, while the relative motion between greenery plants and the camera can cause motion blur^24^. Furthermore, these factors also contribute to the complexity of the background environment in the images, with variations in the size and position of greenery plant areas, ultimately hindering the detection and analysis by networks.

**Figure 1:**
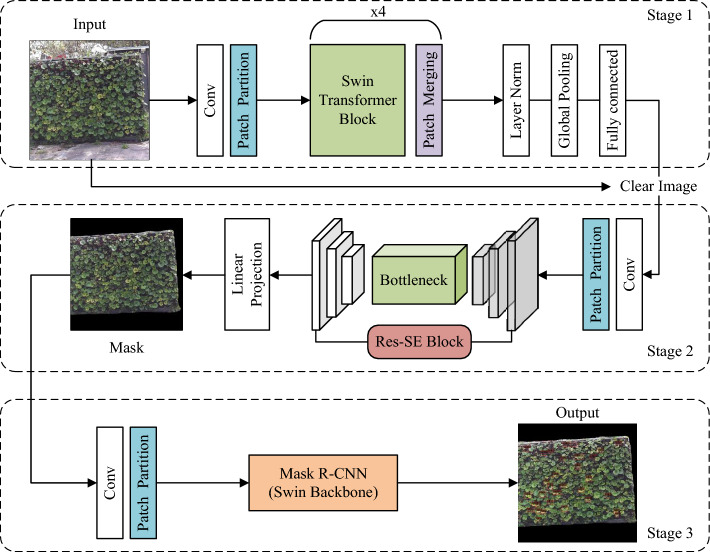
Workflow of the proposed multi-stage progressive detection method.

To address the aforementioned issues, we propose a multi-stage progressive detection method based on Swin Transformer. The overall process structure of the method is shown in Fig. 1, which consists of three stages. Stage 1 constructs a classification network to classify the input vertical greenery plant images into clear and blurry categories and filters out the blurry images. When detecting on low-quality images, the network’s accuracy is low, leading to missed detections and false alarms. Filtering out blurry images effectively improves the detection accuracy of the network. Stage 2 adopts a semantic segmentation network based on the U-shaped structure^25,26^ to segment the vertical greenery areas and background areas in the images. The actual area of the vertical greenery area is calculated based on the segmentation mask, and images with a proportion of vertical greenery area below a certain threshold are filtered out. Such images may result in erroneous analysis of the overall area by the network due to the small effective detection area, failing to accurately reflect the actual growth status of greenery plants. Stage 3 constructs a target detection network to detect water-deficient plants in the images. Additionally, in our multi-stage progressive detection method, we design a waiting mechanism: whenever an image is filtered out, the network waits for a certain period before issuing instructions for re-shooting to avoid re-filtering of the re-shot images. Such a design not only reduces the real-time requirements of the network but also demonstrates strong robustness and stability in practical applications.

In summary, to achieve an end-to-end detection process, we have established different filtering mechanisms at each stage. In the first stage, a classification network is built to filter out low-quality images. In the second stage, a segmentation network is constructed to filter out images with a relatively small proportion of vertical greenery areas and calculate the actual area of vertical greenery. In the third stage, a target detection network is set up to detect water-deficient greenery plants and conduct result analysis. Next, we will provide a detailed introduction to the networks at each stage.

### Classification model

**Figure 2:**
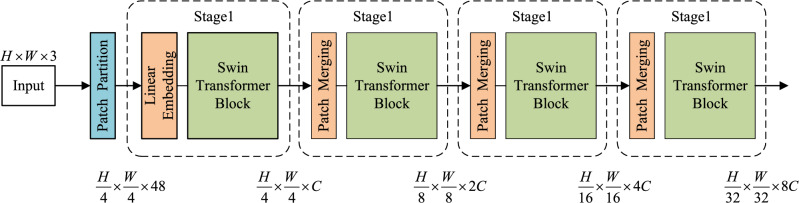
Structure diagram of swin transformer.

The goal of Stage 1 is to achieve clear and blurry classification prediction for vertical greenery images, as shown in Stage 1 of Fig. 1. We appended a LayerNorm layer, a global pooling layer, and a fully connected layer with Sigmoid after the Swin Transformer. Since the Swin Transformer employs Window Multi-Head Self-Attention (W-MSA) for global modeling and feature extraction, and the network initially receives RGB three-channel images, which is not conducive to self-attention computation. Therefore, it is necessary to first perform Patch Partition on the images for block processing. The images after block processing will pass through four stacked hierarchical structures to construct feature maps of different resolutions and extract corresponding image features. Finally, the classification results of the images are output through a fully connected layer with Sigmoid, and images predicted to be clear are input to subsequent stages. The four stacked hierarchical structures are shown in Fig. 2, where Stage 1 consists of a Linear Embedding layer and two Swin Transformer Blocks, while Stages 2 to 4 contain a Patch Merging layer and different numbers of Res-Swin Transformer Blocks.

#### Patch partition

Patch partition divides the input images with a resolution of ${H \times W \times 3}$ into non-overlapping patch collections, with each patch consisting of adjacent ${4 \times 4}$ pixels, and then flattens each patch along the channel dimension. Therefore, the image resolution changes from ${(H \times W \times 3)}$ to ${(\frac{W}{4} \times \frac{H}{4} \times 48)}$ after segmentation by the Patch Partition layer.

#### Patch merging

Patch merging is used to reduce the number of patches and alter the feature dimension. The input feature map is divided into four patches based on neighboring pixels, and then the pixels at corresponding positions on each patch are merged. Subsequently, these four patches are concatenated along the dimension and subjected to a LayerNorm layer. Following this procedure, the resolution of the feature map is halved, while the dimension increases by a factor of 4. Finally, a fully connected layer is applied to perform linear transformation in the depth direction of the feature map, increasing the depth of the feature map from *C* to 2*C* .

**Figure 3:**
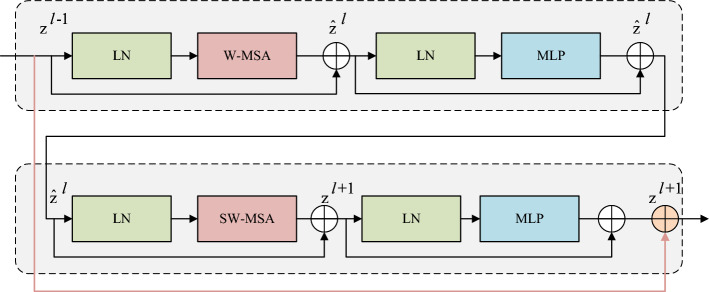
Structure diagram of residual swin transformer block.

#### Res-swin transformer block

As the depth of deep learning network models continues to increase, networks are prone to overfitting and network degradation issues, thereby limiting the improvement in network accuracy. To address this, we introduce the residual idea^27^ into the Swin Transformer Block, connecting the head and tail of the Swin Transformer Block with a shortcut connection. We utilize an identity mapping to prevent the phenomenon of gradient disappearance in the network and simultaneously enhance the model’s generalization ability.

**Figure 4:**
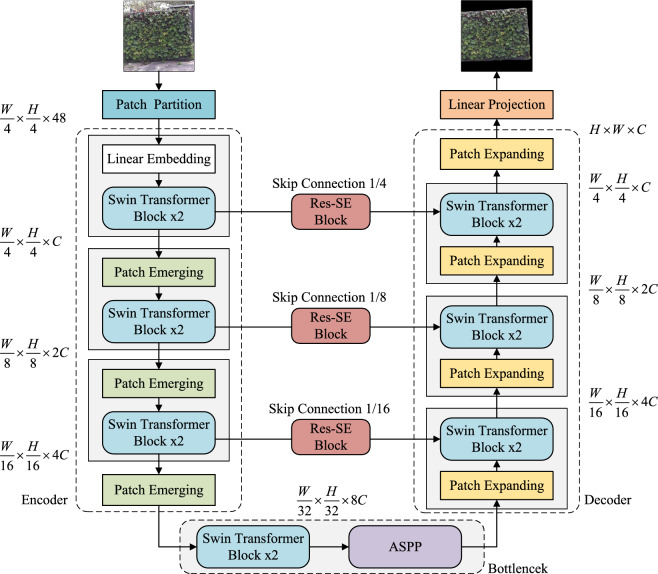
The network structure diagram of improved Swin-Unet.

The structure of the Res-Swin Transformer Block is illustrated in Fig. 3, primarily composed of LayerNorm (LN), Multi-head Self Attention module (MSA), and Multi-Layer Perceptron (MLP). To diffuse attention across different windows, MSA needs to simultaneously utilize W-MSA and SW-MSA, thus Res-Swin Transformer Blocks must be alternately used in pairs. LayerNorm is applied before each MSA and MLP, aiming to normalize different channels of the same sample to maintain the stability of data feature distribution. MLP is a two-layer perceptron with a GELU non-linear activation function, which can reproject features and enhance the network’s non-linear learning capability.

### Semantic segmentation model

The Stage 2 is a U-shaped semantic segmentation network based on Swin-Unet^26^, and the overall architecture of the network is illustrated in Fig. 4, primarily composed of an encoder, bottleneck, decoder, and skip connections.

#### Encoder

The input image is first divided into equally sized, non-overlapping patches through Patch Partition. These transformed patches are then fed into a Transformer-based encoder to learn deep features of the image and generate hierarchical representations. Specifically, the features of the input patches are linearly mapped through Linear Embedding, resulting in an output size of${(\frac{H}{4} \times \frac{W}{4} \times C)}$ (where *C* is a hyperparameter). Subsequently, these patches are passed through multiple Swin Transformer Blocks and the Patch Merging layer to perform down-sampling operations similar to CNN, reducing the resolution of the feature maps. The primary role of the Swin Transformer Block is to extract image features. To enable the network to capture richer multi-scale features, we introduce the Atrous Spatial Pyramid Pooling module (ASPP) at the end of the encoder to extract information from different scales, thereby expanding the receptive field.

#### Atrous spatial pyramid pooling (ASPP)

The ASPP module was first proposed by Chen et al.^28^ in DeepLab V2. This module uses dilated convolutions with different rates in parallel to capture multi-scale features of images, thereby acquiring various receptive field characteristics and integrating them. Subsequently, Wu et al.^29^ improved this module to better consider contextual information and enhance the network’s ability to extract detailed features. Therefore, this paper situates the ASPP module at the end of the encoder, aiming to fully extract multi-scale information, expand the receptive field, and facilitate the decoder in restoring detailed information.

**Figure 5:**
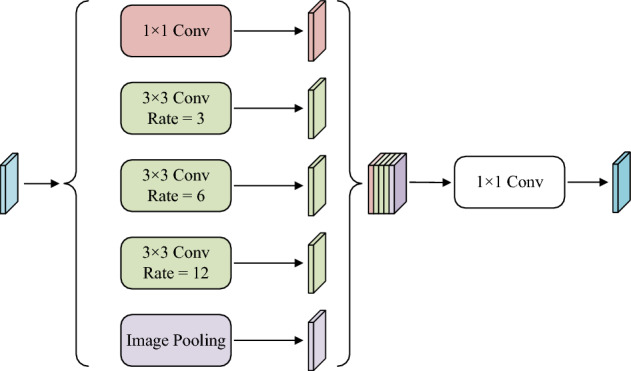
Atrous spatial pyramid pooling module.

The overall structure of ASPP, as shown in Fig. 5, consists of five branches: the first branch utilizes a $1\times 1$ convolution, the second, third, and fourth branches use $3\times 3$ dilated convolutions with dilation rates of 3, 6, and 12, respectively, and the fifth branch employs global average pooling. The image is then restored to its original size using bilinear interpolation. Dilated convolutions, without increasing the model parameter count, have the capability to expand the receptive field of feature maps.

#### Decoder

The decoder is responsible for up-sampling the extracted global image features and gradually restoring the resolution of the feature maps. Each stage consists of Patch Expanding and Swin Transformer Blocks, following a symmetrical structure to the encoder. Patch Expanding enlarges the resolution of the feature map by a factor of 2 through merging features from adjacent dimensions and halving the dimension to achieve up-sampling. The final Patch Expanding expands the length and width of the feature map by a factor of four while keeping the dimension unchanged. To compensate for the loss of detail information during down-sampling, contextual features extracted at the decoder end are skip-connected with multi-scale features at corresponding positions in the encoder, enhancing the restoration of spatial features in the image. Additionally, we introduce a Residual Squeeze-and-Excitation (Res-SE) Block^30^ within the skip connections to emphasize important information in the feature maps and suppress unnecessary information, thereby improving the segmentation accuracy of the image. Lastly, pixel-level segmentation prediction is performed on the vertical greenery area through a Linear Projection layer, which outputs the segmentation mask.

**Figure 6:**
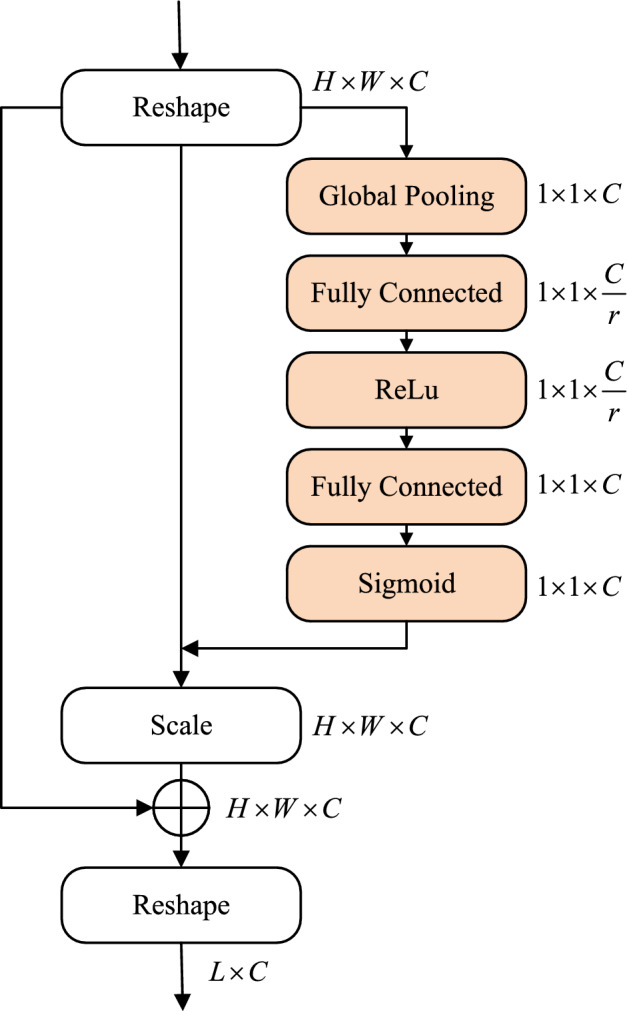
Structure diagram of Res-SE block.

#### Residual squeeze-and-excitation block (Res-SE)

The Res-SE block first reshapes the feature map outputted by the Swin Transformer Block into $\hbox {H}\times \hbox {W} \times \hbox {C}$ dimensions. Subsequently, a series of operations are performed on this feature map, including global average pooling and fully connected layers. The obtained feature map is then multiplied with the original feature map using the Scale operation to create a weighted feature map. Next, through a shortcut connection, the reshaped output of the Swin Transformer Block is added to the weighted feature map, generating a new feature map. Finally, the feature map is resized back to dimensions of $\hbox {L}\times \hbox {C}$ through another reshaping step. This residual attention mechanism neither increases the model’s parameter count nor compromises the original information, effectively preventing overfitting issues. The structure of the Res-SE block is illustrated in Fig. 6.

### Object detection model

**Figure 7:**
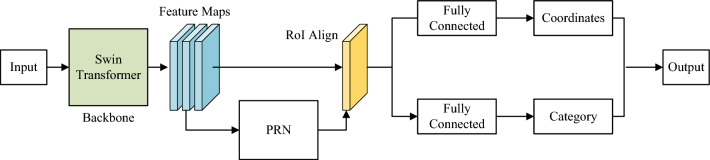
The network structure diagram of mask R-CNN based on swin transformer.

Mask R-CNN is an object detection model in convolutional neural networks. It extends Faster R-CNN by adding a Mask regression branch, capable of simultaneously outputting segmentation prediction, original classification, and bounding box regression tasks. Due to its capability of achieving target pixel-level detection and accurately identifying target contours, Mask R-CNN is suitable for the task of detecting water-deficient plants in this paper. Building upon this, in this phase, Mask R-CNN is used as the foundation, with Swin Transformer employed as the backbone network for feature extraction. The network structure, as illustrated in Fig. 7, primarily consists of four parts: backbone, Region Proposal Networks (RPN), Region of Interest (RoI) Align, and Fully Convolution Nets (FCN). It is noteworthy that we did not include a branch for predicting segmentation masks in the fully connected layer; instead, we focused on the classification and bounding box regression of water-deficient plants. Finally, the ratio of the total area of all predicted boxes to the area of the greenery region was calculated.

### Loss function

The loss function serves as a metric for assessing the consistency between the network output and the expected results. In the detection method proposed in this paper, different outputs are generated at each stage: Stage 1 produces the classification results of the images, Stage 2 outputs segmentation masks, and Stage 3 provides specific prediction information regarding water deficit in plants. Hence, it is imperative to evaluate these outputs separately, calculating classification loss, mask loss, and localization loss. The loss function in this paper comprises three components, with the specific calculation formulas as follows:

1 $$\begin{aligned} {\textrm{Loss}} = {L_{cls}} + {L_{mask}} + {L_{box}} \end{aligned}$$

Where ${L_{cls}}$ denotes the classification loss, ${L_{mask}}$ represents the mask loss, and ${L_{box}}$ signifies the localization loss of the bounding boxes. We employ the Cross-Entropy Loss function for training the classification loss ${L_{cls}}$, Binary Cross-Entropy Loss function for training the mask loss ${L_{mask}}$, and the Smooth L1 Loss function for training the localization loss ${L_{box}}$. The specific calculation formulas are as follows:

2 $$\begin{aligned}{} & {} {L_{cls}} = {\textrm{CELoss}}(y,\hat{y}) = - \sum {{y_i}\log {{\hat{y}}_i}} \end{aligned}$$

3 $$\begin{aligned}{} & {} {L_{mask}} = {\textrm{BCELoss}}(y,\hat{y}) = - [y\log \hat{y} + (1 - y)\log (1 - \hat{y})] \end{aligned}$$

4 $$\begin{aligned}{} & {} {{L_{box}} = {\textrm{smoot}}{{\textrm{h}}_{L1}}({x_i}) = \frac{1}{n}\sum \limits _{i = 1}^n {\left\{ \begin{array}{l} 0.5{\Delta }^2,{\mathrm{}}\left| {{\Delta }} \right| < 1\\ \left| {{\Delta }} \right| - 0.5,{\textrm{other}} \end{array} \right. }} \end{aligned}$$

Where *y* denotes the ground truth, $\hat{y}$ represents the predicted value, *i* stands for anchor index, and ${ \Delta }$ denotes the difference between ${\hat{y}_i}$ and ${y_i}$.

## Experiments and analysis

### Datasets

Due to the high cost of planting vertical greenery plants and the typically good manual care they receive, there are relatively few instances of water deficit and plant death along the roadside, posing a significant challenge in obtaining the required sample images for training purposes^31^. Therefore, this paper focuses on a demonstration area of vertical greenery plants in southwest China. By artificially controlling the watering areas and amounts, plants were induced to exhibit varying degrees of water deficit and death, enabling the capture of images to construct the required greenery plant dataset for this paper.

We utilized a fixed-position intelligent spherical camera to capture images at intervals of 15 minutes every day from March 1 to June 30, 2022, between 07:00 and 19:00. Additionally, to simulate various images captured in real scenarios, we regularly adjusted the lens focal length and shooting angles, capturing images even when the camera rotated. In total, 5808 vertical greenery plant images were collected, featuring diverse angles, sizes, and positions, all with a resolution of $512\times 512$ pixels. These images encompass different weather conditions such as clear skies, cloudy skies, rainy days, and foggy conditions, effectively simulating the variety of vertical greenery plant images one would encounter in real-world settings.

**Figure 8:**
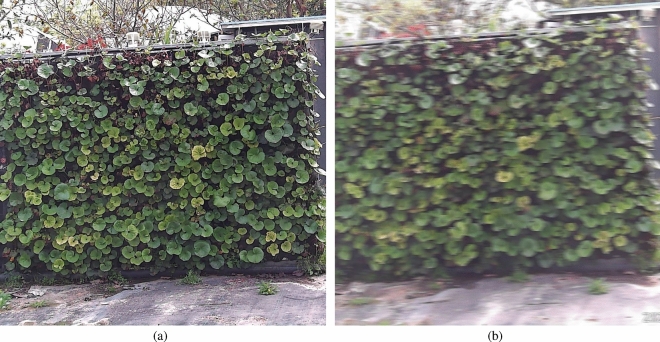
Sample example of a classification dataset: (**a**) clear images; (**b**) blurry images.

**Figure 9:**
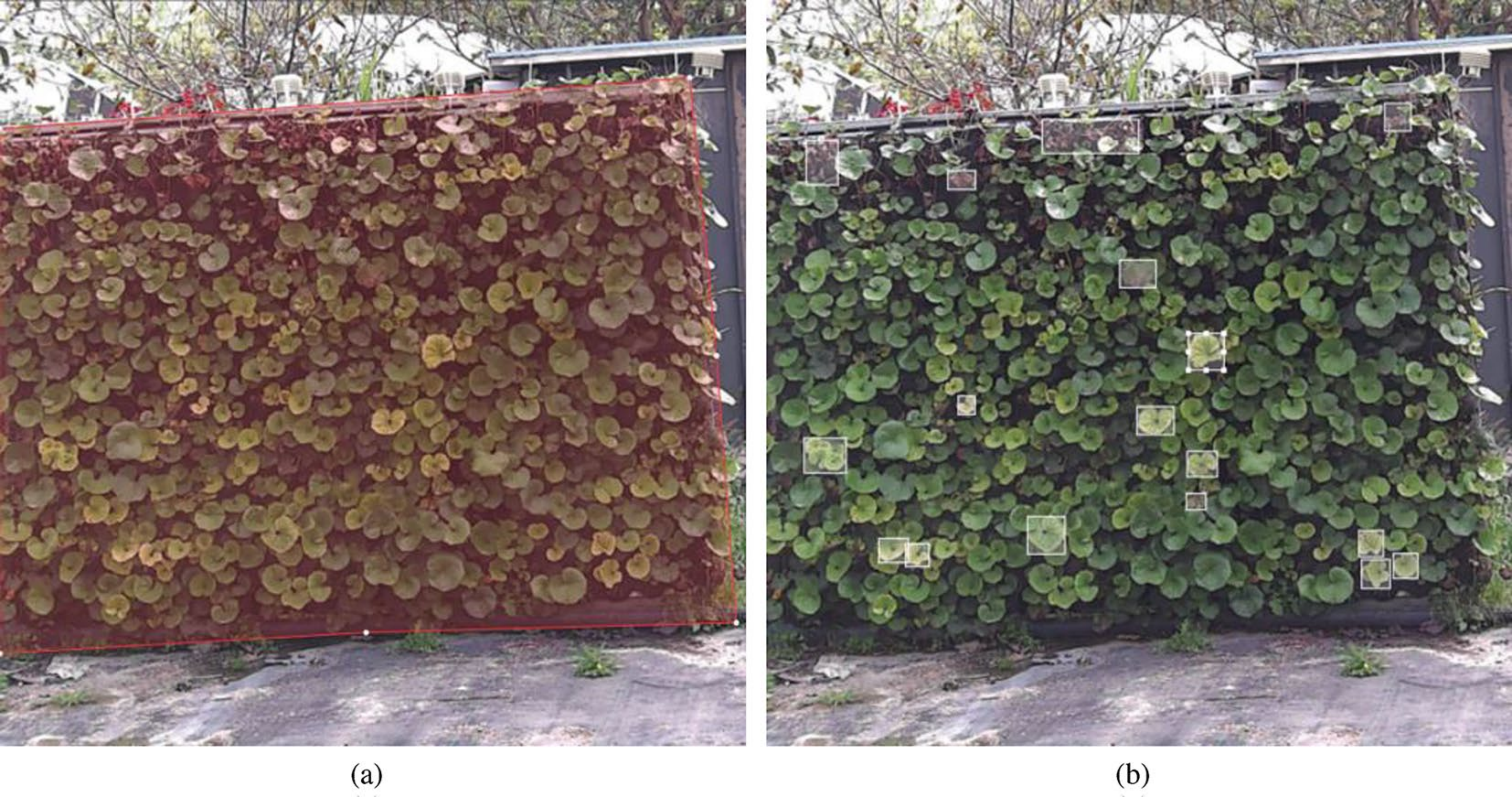
Sample image annotation: (**a**) segmentation dataset annotations; (**b**) detection dataset annotations.

We curated corresponding datasets for each stage. Firstly, a binary classification dataset required for Stage 1 was constructed, where 5808 images were categorized into two classes based on image quality: clear and blurred. Among them, 3980 images belonged to the clear class, while 1828 images were categorized as blurred due to weather, lighting, and blurriness factors. The specific classification is illustrated in Fig. 8. As our objective is to perform image segmentation and detection on these 3980 clear images, lacking corresponding labels necessitated manual image annotation. The vertical greenery areas in the images were annotated using the polygon annotation tool provided by labelme^32^ to generate the segmentation dataset required for Stage 2, as depicted in the annotation example in Fig. 9a. Additionally, the rectangular annotation tool provided by labelimg was utilized to annotate water-deficient and dead plants, generating the object detection dataset, as shown in the annotation example in Fig. 9b. All three datasets were randomly divided into training, validation, and testing sets in an 8:1:1 ratio. The detailed partitioning of the datasets is presented in Table 1.

**Table 1:** The structure of the vertical greenery dataset.

Dataset	Training	Validation	Test
Classification	4646	581	581
Segmentation	3184	398	398
Object detection	3184	398	398

### Model evaluation

The experimental evaluation of the model is mainly conducted from two aspects: detection accuracy and overall performance. We utilize mean Average Precision (mAP) as the evaluation metric for model accuracy, which comprehensively assesses the model’s performance at different IoU thresholds. A higher mAP value indicates that the model possesses stronger high-precision bounding box regression capability, thus enabling more accurate matching between detection results and original labels. Additionally, we evaluate performance using model Parameters, Floating-Point Operations (FLOPs), and Frames Per Second (FPS). Here, Parameters represent the number of parameters in the model; FLOPs are used to measure the model’s complexity and computational load; while FPS indicates the number of images the network can process per second, with its calculation method given in Equation 5.

5 $$\begin{aligned} {\textrm{FPS}} = \frac{1}{{{\textrm{Latency}}}} \end{aligned}$$

Latency refers to the time taken by a network to predict an image, measured in milliseconds (ms).

### Implementation details

The experimental setup in this paper utilized a GeForce RTX 3090 (24GB), PyTorch 1.8.0, and CUDA 10.2. The overall training procedure of the models proceeded as follows: initially, the classification dataset was loaded, and in Stage 1, the classification model was trained, with the weights of the model achieving the best loss being saved upon completion of training. Subsequently, in Stage 2, training of the segmentation model commenced, utilizing the optimal weights saved from Stage 1 as initial weights. Additionally, the network model from Stage 1 was frozen during this stage. The weights achieving the best loss were saved upon completion of training. Following a similar protocol, training for Stage 3 was conducted. The initial weights for Stage 1 were initialized using pre-trained weights from the ImageNet-1K dataset.

The general training parameters for each stage model are as follows: the resolution of input images is $256\times 256$ pixels, the backbone network utilizes the Tiny model of Swin Transformer, the vector dimension *C* is 96, the repetitions of Swin Transformer Blocks are (2, 4, 2, 2), the image patch size is 4 pixels, the training batch size is 4, and the Adam optimizer is employed to update the model parameters. Specifically, the training settings for each task model are as follows: for the classification model, the number of training epochs is 150, the initial learning rate is 5e-5, and the loss function is cross-entropy loss; for the segmentation model, the number of training epochs is 100, the initial learning rate is 5e-5, the loss function is Binary Cross-Entropy Loss, and the filtering threshold for vertical greenery areas is 0.4; for the detection model, the number of training epochs is 100, the initial learning rate is 1e-5, the classification loss function for targets is Cross-Entropy Loss, and the bounding box regression loss function utilizes the Smooth L1 Loss function.

### Experiments results

#### Ablation experiment

To validate the effectiveness of the proposed multi-stage progressive detection method, we sequentially integrated the models of each stage and the Swin Transformer backbone into the baseline model for ablation experiments. To ensure the reliability of the experimental results, all experiments were conducted on the same training set consisting of 3184 images from the object detection dataset in “Multi-stage progressive detection architecture”, with a training duration of 300 epochs. However, due to the absence of low-quality images in this dataset, direct evaluation on the test set (398 images) would not reasonably assess certain experiments. Therefore, we augmented the test set with 200 images classified as blurred in the classification dataset and annotated them in the same manner, resulting in a final set of 598 untrained test images. On this test set, we evaluated the impact of each stage on model performance using evaluation metrics. The experimental results are presented in Table 2.

**Table 2:** Ablation experiment of multi-stage progressive detection method.

Experiment	Stage1	Stage2	Stage3	Swin-T	mAP@[0.5, 0.9]	FLOPs(G)	Params(M)	FPS
1			$\checkmark$		74.3	150.8	55.7	16.7
2	$\checkmark$		$\checkmark$		86.6	184.4	94.4	13.4
3		$\checkmark$	$\checkmark$		80.7	213.5	118.1	10.8
4	$\checkmark$	$\checkmark$	$\checkmark$		90.1	150.8	153.2	8.1
5	$\checkmark$	$\checkmark$	$\checkmark$	$\checkmark$	93.5	233.2	139.8	9.4

We used the Mask R-CNN^12^ model with only pre-trained weights as the baseline model, achieving an mAP of 74.3% on the test set . Experiments 2 and 3 adopted a two-stage detection method, where the Stage 1 classification model and Stage 2 segmentation model proposed in this paper were added before the baseline model. The experimental results revealed that both sets of experiments showed improvement in mAP compared to the baseline model, with increases of 12.3% and 6.4%, respectively. Experiment 2 exhibited a more pronounced enhancement, attributed to the effective filtration of low-quality images by the added classification model, leading to a notable increase in the overall method’s accuracy. Importantly, Experiment 2 had lower parameter and computational overhead increases compared to Experiment 3. This phenomenon can be explained by the fact that the classification model does not require up-sampling of feature maps, thereby reducing network parameters and computational load. In Experiment 4, both the classification and segmentation models were integrated into the baseline model, forming a three-stage detection method. This method achieved the most significant improvement in mAP, increasing by 19.2%, validating that the proposed multi-stage method effectively enhances the accuracy of vertical greenery plant image detection.

Building upon Experiment 4, we incorporated the Swin Transformer as the backbone network in the detection model to further validate its effectiveness, as shown in Table 2.. In comparison to Experiment 4, there was a 4.6% increase in mAP, a 16% rise in the performance metric FPS, and a decrease of 13.4G FLOPs. This enhancement can be attributed to the Swin Transformer segmenting the image into multiple patches for sequential modeling and extracting multi-scale features, thereby integrating more detailed information into the base features. Additionally, it utilizes windowed attention mechanisms to reduce the interdependencies in computations at each position. Therefore, employing Swin Transformer as the backbone not only effectively enhances the accuracy of object detection but also reduces network computational load to some extent, accelerating model inference speed.

#### Comparative experiment

**Table 3:** Comparative experimental results of different detection models.

Model	mAP@[0.5, 0.9]	FLOPs(G)	Params(M)	FPS
Mask R-CNN^12^	74.3	150.8	55.7	16.7
YOLOv7^33^	76.2	82.5	45.7	32
DETR^34^	79.7	87.6	41.4	29
Deformable DETR^35^	84.3	101.5	54.4	26.7
Ours	93.5	233.2	139.8	9.4

To verify the superiority of the multi-stage progressive method proposed in this paper, we conducted comparative experiments with traditional object detection models such as Mask R-CNN^12^ and YOLOv7^33^, as well as Transformer-based end-to-end object detection models like DETR^34^ and Deformable DETR^35^. The experimental results are presented in Table 3.. Apart from varying the network models, all other experimental conditions remained consistent, including the datasets used and the training methods. Table 3. demonstrates that the multi-stage detection method proposed in this paper exhibits outstanding performance in mAP, achieving 93.5%. This represents significant improvements of 19.2%, 17.3%, 13.8%, and 9.2% compared to other models, showcasing remarkable enhancement effects. Although the multi-stage detection method has seen an increase in FLOPs, Params, and FPS to 233.5G, 139.8M, and 9.4, respectively, by replacing Swin Transformer as the backbone network, we significantly reduce the model computational load, enabling the model inference speed to be comparable to traditional two-stage object detection models. The multi-stage progressive detection method proposed in this paper meets real-time requirements and significantly boosts detection accuracy by 19.2%, achieved through an increase of 82.4G FLOPs and 84.1M parameters.

**Figure 10:**
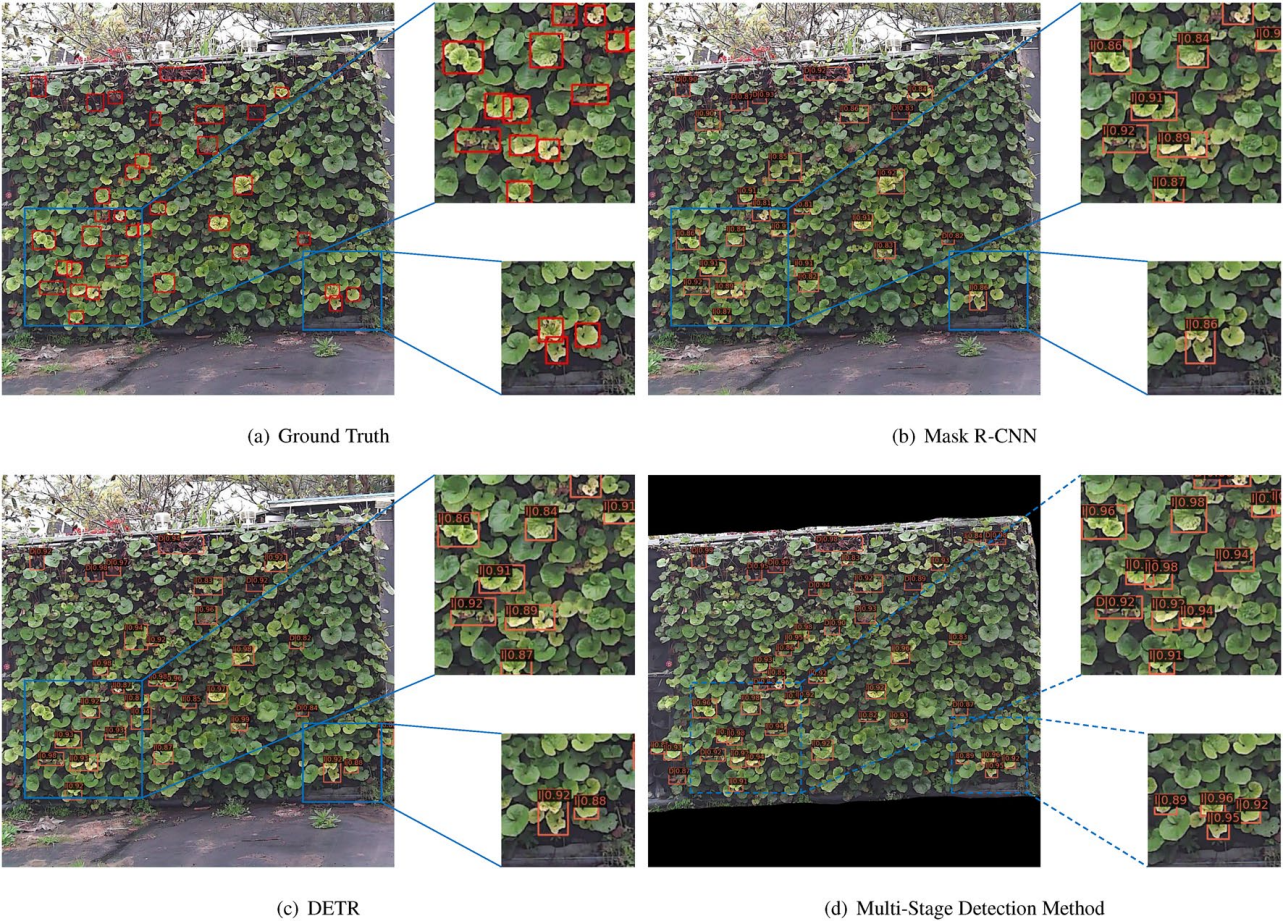
Comparison of different models through visualization. **(a)** Ground truth, **(b)** mask R-CNN, **(c)** DETR, **(d)** multi-stage detection method.

We selected a representative sample image from the clear dataset for visualizing the test results in order to evaluate the effectiveness of the method more intuitively. Figure 10 compares the results on this sample image, where Fig. 10a represents the ground truth, while Fig. 10b–d respectively display the detection results of Mask R-CNN, DETR, and our method. By comparing the visualized images, it can be noted that the detection performance of Mask R-CNN is the poorest, exhibiting significant issues of missed detections and false alarms. Although the miss rate of DETR is lower than that of Mask R-CNN, it still tends to merge multiple overlapping water deficit plants into one entity and mistakenly detects plants in external non-vertical greenery areas. In contrast, our method is capable of distinguishing overlapping plant targets, with no missed detections. Additionally, by introducing masks in the second stage for non-vertical greenery areas, the detection focus is directed towards the interior of the vertical greenery plants, effectively reducing the false alarm rate. In conclusion, our proposed multi-stage progressive detection method demonstrates the best performance.

## Conclusion

This paper proposes a multi-stage progressive detection method based on Swin Transformer for addressing practical issues in engineering applications. The method integrates three networks for image classification, image segmentation, and object detection, employing a multi-stage architecture to incrementally process images, thereby overcoming the problem of low detection accuracy in existing models under conditions such as low-light nighttime and motion blur. To further enhance detection accuracy and efficiency, each stage adopts Swin Transformer as the backbone network, utilizing its position-based self-attention mechanism to extract multi-scale features of images, thereby improving the overall efficiency of the network and reducing computational complexity. Some positions utilize Res-Swin Transformer Blocks to retain the original feature information of images while effectively preventing overfitting issues. In the image segmentation of stage 2, the ASPP module is introduced to fuse image features of different scales and enlarge the receptive field; simultaneously, a residual attention mechanism is incorporated in the skip connections to enhance important feature information and diminish redundant background features. The experimental results demonstrate that compared to other models, the multi-stage progressive detection method achieves the highest mAP, with visualized results closer to the ground truth, confirming the effectiveness and superiority of our method.

While multi-stage methods have achieved significant improvements in detection accuracy, their integration of multiple networks results in increased overall model complexity and computational requirements, thus imposing higher demands on deployment devices. Therefore, in the future, we aim to further optimize the model, reduce complexity, and consider migrating time-consuming classification and segmentation tasks to the cloud to lessen the demands on deployment devices and reduce delays during detection. Overall, the multi-stage progressive detection method proposed in this paper overcomes the limitations of traditional detection methods, offering an easily deployable and practical automated method for detecting vertical greenery plants. This method also holds broad application value and can be extended to other areas such as crop pest detection and fruit ripeness detection.

## Data Availability

The datasets generated and analysed during the current study are available in the Google Drive(https://drive.google.com/file/d/11mNweBe2WVvULplaKawVAcEA0CBFQCA4/view?usp=drive_link).
